# Supplementary material for: Axon-like active signal transmission
Source: Nature. 2024 Sep 11;633(8031):804–10. doi: 10.1038/s41586-024-07921-z (PMC11424471; doi:10.1038/s41586-024-07921-z)
Supplement: Supplementary file 1 — Supplementary Notes 1–3 and Figs. 1–22. [file 41586_2024_7921_MOESM1_ESM.pdf]

nature portfolio

https://doi.org/10.1038/s41586-024-07921-z

Supplementary information

Axon-like active signal transmission

In the format provided by the authors and unedited

Axon-like Active Signal Transmission

Timothy D. Brown[1], Alan Zhang[1], Frederick U. Nitta[1,2], Elliot D. Grant[1], Jenny L. Chong[3], Jacklyn Zhu[1], Sritharini Radhakrishnan[1], Mahnaz Islam[1,2], Elliot J. Fuller[1], A. Alec Talin[1], Patrick J. Shamberger[3], Eric Pop[2], R. Stanley Williams[1,3], Suhas Kumar[1]

[1] Sandia National Laboratories, Livermore, CA, USA
[2] Stanford University, Stanford, CA, USA
[3] Texas A&M University, College Station, TX, USA

SUPPLEMENTARY INFORMATION

CONTENTS

Section	Page
1. Theory: Edge of chaos, poles and zeros, and phase shifts	2
2. Compact model	6
3. Additional figures (referenced in the main text or in the Methods)	12

1. Theory: Edge of chaos, poles and zeros, and phase shifts

1.1. Linear stability analysis for sinusoids

Questions of stability, instability, and semi-stability are most directly treated through the analysis of complex transfer functions. To keep the discussion physically grounded, we review the fundamentals for using complex functions for stability analysis in a slightly different way than encountered in textbooks.

Any decaying, growing, or constant-amplitude sinusoid signal $x(t)$ can be represented as a complex number $s = \alpha + j\omega$, with each component of s related to the growth constant and frequency of the sinusoidal $x(t)$. This representation of real sinusoids as complex numbers is encoded as

$$x(t) = A\exp(\alpha t) \times \cos(\omega t) \leftrightarrow s = \alpha \pm j\omega. \qquad \text{Eqn. (S1)}$$

For example, we distinguish decaying sinusoids ($\alpha < 0$) from growing sinusoids ($\alpha > 0$) and constant amplitude sinusoids ($\alpha = 0$). Since α is the real part of the complex representation of the sinusoid, i.e., $\alpha = \Re(s)$, $\Re(s)$ plays a central role in describing the rate of growth (or decay) of the amplitude of $x(t)$.

Assuming $x(t)$ to be a sufficiently small perturbation on a steady state, the action of any circuit on an input, $x(t)$, produces a scaled and phase shifted version of it as its output, $y(t)$. For example, the $LaCoO_3$ devices in this work transformed a small constant-amplitude AC sinusoidal voltage into a constant-amplitude and phase-shifted AC current at the same frequency. Hence the output of the circuit is $y(t) = G \times x(t + \Delta\phi)$, with gain G being the ratio of the output and input amplitudes, and $\Delta\phi$ being their relative phase shift. Equivalently, the action of the system transforms the input according to the linear operator Ψ:

$$\Psi: x \to y = (G, \Delta\phi) \circ x, \qquad \text{Eqn. (S.2)}$$

where $(G, \Delta\phi) \circ$ signifies "phase shift by $\Delta\phi$ and increase amplitude by G." Usefully, when the sinusoid signals are encoded as complex numbers, Ψ reduces to a simple multiplication $\times$ of complex numbers:

$$\Psi: s \longrightarrow w = H \times s, \qquad \text{Eqn. (S3)}$$

where w is the complex encoding of $y(t)$, and H is the complex number with magnitude $G = |H|$ and phase shift $\Delta\phi = \angle H$. Generally, Ψ acts differently on different inputs s, so that H is a function of s. The complex function $H(s)$ is termed as the transfer function for the circuit under study. The foregoing implies a simple and intuitive interpretation of the complex transfer function $s \to w$: for any input sinusoid with growth constant α and frequency ω, the output is the same, but scaled by $|H(s)|$ and phase-shifted by $\angle H(s)$.

For sufficiently small $x(t)$, $H(s)$ takes the form of a ratio of two polynomials. An especially important aspect of $H(s)$ is its poles, s_{p}, where the denominator is zero. The gain near the poles is especially high

$G \gg 1$, so even if $x(t)$ consists of many spectral components, $y(t)$ will be dominated only by the components near the poles: the poles characterize the natural response of the circuit. If even just one of the poles is in the right half of the complex plane (RHP, $\Re(s)$>0), then the output is dominated by an exponentially growing component ($\alpha_{\mathrm{p}} > 0$), and the circuit is unstable. It is stable otherwise ($\Re(s_p) \leq 0$).

1.2. Edge of chaos, semi-stability, and phase shifting

Just as the poles of $H(s)$ are defined as points where its denominator becomes zero, its "zeros" are points where its numerator becomes zero. At first, there appears to be little use in analyzing the zeros of $H(s)$, since they have no direct bearing on stability or instability. However, from the beginning, this analysis has assumed a well-defined input $x(t)$ and output $y(t)$, i.e., the signal $x(t)$ has been arbitrarily designated as the input. But the analysis is symmetric and can be equally performed with the input and output interchanged. In this case, the effect of the circuit on its input is inverted, and is represented in complex numbers as

$$\Psi^{-1}: w \to s = \frac{1}{H(s)} \times w. \qquad \text{Eqn. (S4A)}$$

In other words, after interchanging the input and output, the transfer function becomes its own reciprocal,

$$H^{-1}(s) = \frac{1}{H(s)}. \qquad \text{Eqn. (S4B)}$$

Since $H(s)$ is a ratio of polynomials, the zeros of $H(s)$ are the poles of $H^{-1}(s)$, and therefore the zeros of $H(s)$ determine the stability of the circuit after interchanging its input and output. But the poles and zeros of $H(s)$ are generally independent of one another; hence, the stability of the circuit before and after interchanging its input and output are also independent. In particular, the circuit may be such that Ψ is stable but Ψ^{-1} is unstable; the stability of the circuit is therefore conditional on the input used to query it. We have termed this situation as semi-stability. From the foregoing discussion, a semi-stable state is characterized by either $H(s)$ or $H^{-1}(s)$ having at least one zero in the RHP, but all poles in the left half-plane (LHP), i.e., the overall configuration of poles and zeros straddles the imaginary axis. In contrast, a totally stable state would necessarily have all poles and zeros of both $H(s)$ and $H^{-1}(s)$ in the stable LHP.

In experiments it is common to measure the gain and phase shift for sinusoids with constant amplitude and varying frequency ($s = 0 + j\omega$). In this way, the stability properties of the poles of $H(s)$ are probed indirectly through the one dimensional transect $H(j\omega)$. Given restrictions that apply to the present $LaCoO_3$ devices in the main text, namely that their electrical conductivity is dominated by temperature and approximately independent of voltage, it has been shown that the phase shifts for constant-amplitude

sinusoids $\angle H(j\omega)$ present an unusual feature. Within some limited bandwidth $0 \leq \omega \leq \omega_{\text{max}}$, the phase shifts $\Delta\phi$ lie within the second or fourth quadrants of the unit circle, $\frac{\pi}{2} < |\Delta\phi| \leq \pi$. This phase shifting condition, $\frac{\pi}{2} < |\angle H(j\omega)| \leq \pi$, together with the requirement that the pole of $H(s)$ is stable, is what Chua has defined as Edge of Chaos (EoC). Thus, as far as the work in the main text is concerned, semi-stability and edge of chaos occur together, and generate super-quadrature phase shifts $\frac{\pi}{2} < |\Delta\phi| \leq \pi$ that we have verified through experimental characterization in the main text.

We have illustrated this mutual dependence of semi-stability, input-output inversion, edge of chaos, and super quadrature phase shifts in Supplementary Fig. 1. The figure panels were generated from the compact model we developed to model the experimental data (detailed in section 7), and so, instead of being merely schematic, they reflect the real experimental conditions and measured data. Each of the two columns of the plot (panels (a)-(c) and (d)-(f)) corresponds to computations with respect to the compact model, for a positive differential resistance bias at 2.0 mA, versus a negative differential resistance bias at 3.5 mA. Within each column of the plot, we have shown the two transfer functions relating the current and voltage, i.e., impedance Z ($\hat{v} = Z \times \hat{\imath}$) and its inverse, admittance Y ($\hat{\imath} = Y \times \hat{v}$), where $\hat{\imath} \leftrightarrow i(t)$ and $\hat{v} \leftrightarrow v(t)$. The transfer functions are plotted in the complex plane ($\alpha + j\omega$) using color to represent phase, and contours to represent points with constant magnitude. For example, the 1×10^3 contour in Supplementary Fig. 1a connects the current signals $i(t) \leftrightarrow \alpha + j\omega$ for which the gain is 1×10^3 V/A.

In moving from the $Z(s)$ plots (Supplementary Fig. 1a,d) to the $Y(s)$ plots (Supplementary Fig. 1b,e), the roles of current and voltage as input and output have been swapped, H becomes H^{-1}, and as a result, the pole (black) and zero (white) exchange positions. For the 2.0 mA data (Supplementary Fig. 1a-c), the input-output interchange has no effect on the stability, so both Z and Y at this bias are stable (both the pole and the zero are in the LHP in both cases). In contrast, for the 3.5 mA data (Supplementary Fig. 1d-f), interchanging the input and output moves the pole from the LHP to the RHP, and so, Z is stable but Y is *unstable* at this bias. This is the phenomenon of semi-stability. Finally, in Supplementary Fig. 1c,f, the impedance phase shifts of constant amplitude current and voltage sinusoids are evaluated by projecting Z onto the imaginary axis, $s = j\omega$. For the 2.0 mA data, the phase shifts are always sub-quadrature $|\Delta\phi| < \pi/2$. In contrast, for the 3.5 mA data, there is a limited bandwidth $0 \leq \omega \leq 1590$ rad s^{-1} for which the phase shifts are super-quadrature $\pi/2 \leq |\Delta\phi| < \pi$, and thus, it is Edge of Chaos. In this way, one can intuitively observe the inherent links between semi-stability, edge of chaos, and super-quadrature phase shifts.

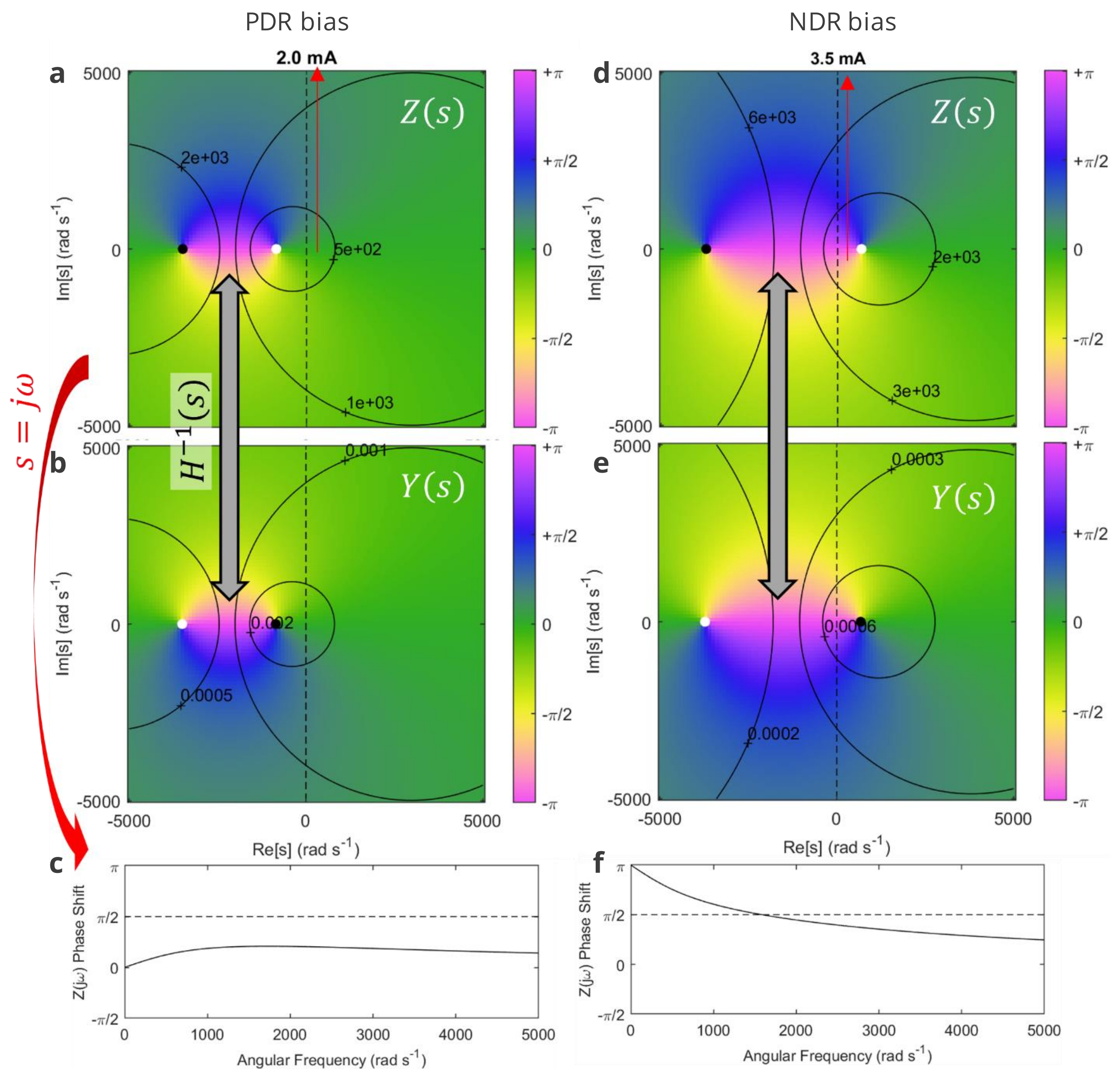

Supplementary Fig. 1: Complex transfer functions, semi-stability, and super-quadrature phase shifts. Magnitude-phase plots of (a) the impedance $Z(s)$ and (b) admittance $Y(s)$ transfer functions, and the corresponding frequency-dependent phase shift for impedance on the imaginary axis, $\angle Z(j\omega)$, for a PDR bias (2 mA). Poles are marked with solid black dots, zeros with solid white dots, and the Im axis is marked with a vertical dashed line, with the red arrow next to it. Impedance, admittance, and phase shifts were computed using the compact model developed in section 7. No super-quadrature phase shift is observed in (c). (d)-(f) are plots similar to (a)-(c), but for an NDR bias of 3.5 mA. For the 3.5 mA data, Z is stable, Y is unstable, and super-quadrature phase shifts are observed within a limited bandwidth.

For completeness, the procedure for determining the transfer function (either impedance $Z(s) = v/i$ or admittance $Y(s) = i/v$) is discussed in detail elsewhere [*Advanced Materials 35, 2205451 (2023); Applied Physics Reviews 9, 011308 (2022)*], but in brief, the transfer functions at a steady state (T, I, V) are:

$$Z(s;T) = \frac{1}{G_{\mathrm{el}}(T)} \times \left(\frac{s - s_1(T)}{s - s_2(T)}\right), \qquad \text{Eqn. (S5A)}$$

$$Y(s;T) = \frac{1}{Z(s;T)}, \qquad \text{Eqn. (S5B)}$$

$$s_{1,2}(T) = \frac{G_{\mathrm{th}}(T)}{C_{\mathrm{th}}(T)} \times \left(\left(\frac{G_{\mathrm{el}}'(T)}{G_{\mathrm{el}}(T)} \pm \frac{G_{\mathrm{th}}'(T)}{G_{\mathrm{th}}(T)}\right) \times (T - T_0) \pm 1\right), \qquad \text{Eqn. (S5C)}$$

where $G_{\mathrm{el}}(T)$ and $G_{\mathrm{th}}(T)$ are the device's temperature-dependent electrical and thermal conductances, and $C_{\mathrm{th}}(T)$ is its heat capacity, and x' denotes $\mathrm{d}x/\mathrm{d}T$. The table below contains the values of these parameters evaluated using the compact model corresponding to the transfer function plots. Observe that the units of Z, Y, and s are Ω, S, and rad s^{-1}, respectively; these are all real, physically measurable quantities.

Table S1: Table of pre-factors and pole / zero frequencies for transfer function plots

Bias Current [mA]	$G_{\mathrm{el}}(T)$ [S]	$G_{\mathrm{th}}(T)$ [W K^{-1}]	$C_{\mathrm{th}}(T)$ [J K^{-1}]	$s_1(T)$ [rad s^{-1}]	$s_2(T)$ [rad s^{-1}]
2.0	1.16×10^{-4}	7.10×10^{-4}	5.11×10^{-7}	-8.54×10^{2}	-3.50×10^{3}
3.5	2.04×10^{-4}	7.69×10^{-4}	5.11×10^{-7}	$+6.77\times10^{2}$	-3.73×10^{3}

2. Compact model

2.1. Compact model summary

A compact model is a representation of device behavior as a nonlinear dynamical system, i.e., as a solution to a system of nonlinear differential equations. For the $LaCoO_3$ test structure, a compact model was developed describing it as a parallel network of a capacitor and a first-order electro-thermal memristor. The accompanying equations describing the model are

$$\frac{\mathrm{d}T}{\mathrm{d}t} = \frac{1}{C_{\mathrm{th}}} \times \left(v^2 \times G_{\mathrm{el}}(T) - (T - T_0) \times G_{\mathrm{th}}(T)\right), \qquad \text{Eqn. (S8)}$$

$$\frac{\mathrm{d}v}{\mathrm{d}t} = -\frac{1}{C_{\mathrm{el}}} \times \left(v \times G_{\mathrm{el}}(T) - (I_{\mathrm{DC}} + i_{\mathrm{AC}})\right). \qquad \text{Eqn. (S9)}$$

Where T and v are the instantaneous temperature and voltage of the device; other relevant definitions are in Table S2. Equations S8-S9 are derived from conservation of heat and charge. The motivation and context

for the model to follow are similar to our previous work [*Advanced Materials 35, 2205451 (2023); Applied Physics Reviews 9, 011308 (2022)*], and a comprehensive account will be given in future work. For this supplementary section we will simply present all mathematical details needed to implement the model in any numerical solver.

The temperature-dependent electrical conductance function was modeled as two piece-wise Arrhenian functions at low and high temperature, with a logistic interpolation between their logarithms:

$$G_{\mathrm{el}}(T) = \frac{A}{L} \times \sigma_{\mathrm{interp}}, \qquad \text{Eqn. (S10A)}$$

$$\log \sigma_{\mathrm{interp}} = \left(1 - s(T)\right) \times \left(\log \sigma_1^{\mathrm{Arrh}}\right) + s(T) \times \left(\log \sigma_2^{\mathrm{Arrh}}\right), \qquad \text{Eqn. (S10B)}$$

$$\sigma_{1,2}^{\mathrm{Arrh}} = \sigma_{1,2}(T_0) \times \exp\left(-\frac{E_{1,2}}{k_B} \times \left(\frac{1}{T} - \frac{1}{T_0}\right)\right), \qquad \text{Eqn. (S10C)}$$

$$s(T) = \left(1 + \exp\left(-\left(\frac{T - T_c}{\delta T}\right)\right)\right)^{-1} \qquad \text{Eqn. (S10D)}$$

The temperature-dependent thermal conductance function was modeled as a sigmoid interpolant between two constant values.

$$G_{\mathrm{th}}(T) = K_0 + (K_1 - K_0) \times \left(1 + \exp\left(-\left(\frac{T - T_{\mathrm{m}}}{\partial T}\right)\right)\right)^{-1}. \qquad \text{Eqn. (S11)}$$

The electrical and thermal capacitances of the circuit were set as constants. Finally, the input current to the model consisted of a varied DC component (as in experiments), summed with a 10 µA constant amplitude AC component at varied frequencies:

$$i_{AC} = 1 \times 10^{-5} \times \cos(2\pi f t). \qquad \text{Eqn. (S12)}$$

The parameters' names, values, and units are in the tables below. Together with the model equations above, this fully reproduces the compact model and can be implemented on any numerical integrator. All model parameters were fixed despite varying the DC bias current and AC frequency, since these were the only experimental dependent parameters as well.

Table S2: Electrical Conductance Model

Parameter	Description	Fit Value	Units
T_0	Ambient temperature for experiments	300	K
k_B	Boltzmann constant	8.617×10^{-5}	eV/K
σ_1	Low temperature electrical conductivity at T0	89.56	S/m
σ_2	High temperature electrical conductivity at T0	2302.24	S/m

$E_{a,1}$	Low temperature activation energy	0.178	eV
$E_{a,2}$	High temperature activation energy	0.155	eV
T_c	Critical temperature; sigmoid = 50%	503.2	K
δT	Width of sigmoid	50.84	K
A/L	Device dimensions prefactor	4.3E-7	m

Table S3: Thermal Conductance Model

Parameter	Description	Fit Value	Units
K_0	Low-temperature constant factor, (pre-sigmoid)	7.77×10^{-5}	W/K
K_1	Low-temperature constant factor, (post sigmoid)	7.70×10^{-4}	W/K
T_{m}	Midpoint temperature; sigmoid = 50%	334.8	K
∂T	Width of sigmoid	6.87	K

Table S4: Fixed electrical and thermal capacitances

Parameter	Description	Fit Value	Units
C_{el}	Effective circuit electrical capacitance	1.0×10^{-8}	F
C_{th}	Device heat capacity	5.11×10^{-7}	J/K

2.2. Development of the compact model

We give a brief review of the overall fitting process for the model parameters. The piecewise-Arrhenian compact model was fit to the temperature-dependent electrical conductivity $\sigma(T)$ in main Fig. 2b (Supplementary Fig. 2a). All of the model parameters were fit with automated optimization routines, either least-squares fitting to the low and high conductivity models, or Matlab's nlinfit optimizer for the sigmoid interpolant. Next, the electrical conductivity data were combined with the quasistatic current-voltage data on the test device (main Fig. 2d). This comprised several sub-steps:

First, the power $P = I \times V$ and electrical conductance $G_{\mathrm{el}} = I/V$ were calculated at every point on the quasistatic IV curve.

Second, the active device aspect ratio A/L was self-consistently determined such that $A/L \times \sigma(300\ \mathrm{K})$ was equal to the recorded I/V near the start of the IV curve (i.e., approximating $\lim_{I\to0} I/V$). Although the device dimensions are nominally known, and they deviate by < 5% from the active dimensions, this is a necessary step to self-consistently merge the electrical conductivity and quasi-static data, which prevents accidental division by zero or near-zero in later steps. After this step, the full $G_{\mathrm{el}}(T)$ model was complete.

Third, for each point on the quasistatic curve, the measured electrical conductance was converted into a computed temperature, by numerically inverting the electrical conductance model. Specifically, the model was used to generate a selection of (T, G_{el}) points, then pairs were inverted as (G_{el}, T), and then used as an

interpolation grid for the measured I/V from the quasistatic data. Thus, each IV point was assigned a derived temperature by numerically approximating the inverse model $G_{\mathrm{el}} \rightarrow T$.

Fourth, the steady state condition,

$$G_{\mathrm{th}}(T) = \frac{P(I,V)}{T(I,V) - T_0}, \qquad \text{Eqn. } (S13)$$

was applied to the derived temperatures to determine the thermal conductance at each point.

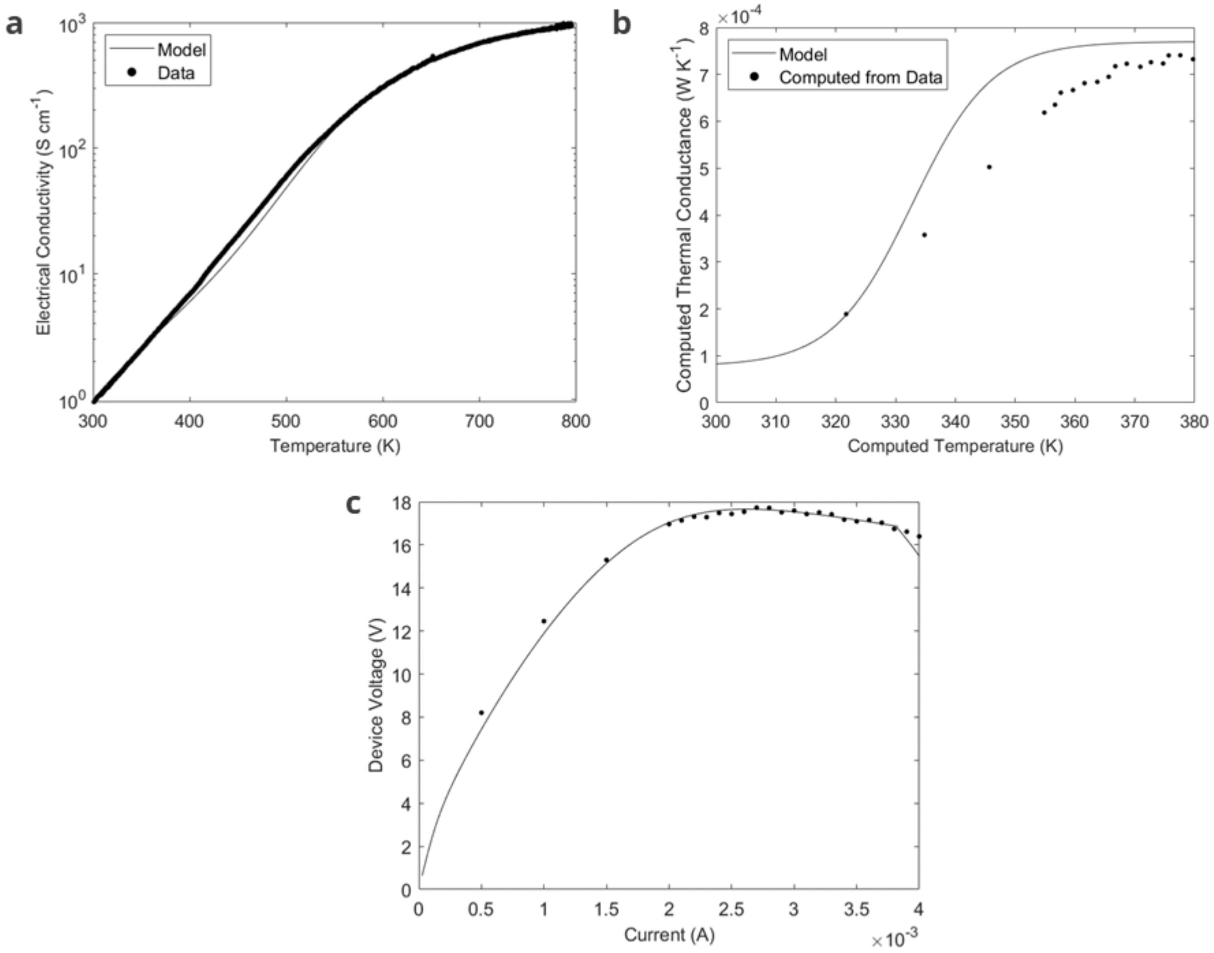

Supplementary Fig. 2: Data and models for electrical and thermal properties of $LaCoO_3$ devices. (a) Temperature dependent electrical conductivity data were fit directly to the piecewise Arrhenian model described in the text. (b) Although never measured directly, a temperature-dependent thermal conductance curve was generated as an intermediate step using the IV data and steady-state conditions. (c) With this intermediate step, the quasi-static current-voltage data could be fit systematically.

Thus, the fitted electrical conductivity model, the measured quasistatic IV data, and the steady state condition were combined to self-consistently compute $G_{\mathrm{th}}(T)$. The shape of the derived $G_{\mathrm{th}}(T)$ curve

motivated the sigmoidal model (Supplementary Fig. 2b). A more accurate fit to $G_{\mathrm{th}}(T)$ was initially derived, but the model was not well fit to the IV curve. Since the IV curve was directly measured by experiment; whereas the $G_{\mathrm{th}}(T)$ curve was only indirectly computed, we prioritized a more accurate fit to the IV data (Supplementary Fig. 2c) at the expense of the accuracy of the $G_{\mathrm{th}}(T)$ curve. We include the $G_{\mathrm{th}}(T)$ curve and its fit here for completeness, and to illustrate the general procedure of computing a (theoretical) $G_{\mathrm{th}}(T)$ curve by combining data sets, then using the $G_{\mathrm{th}}(T)$ fit as a starting point to obtain an accurate fit to measured $I - V$ data. We find this intermediate step to be more systematic than trying to fit the IV curve directly, without any idea of the thermal properties. The thermal capacitance C_{th} divided by $G_{\mathrm{th}}(T)$ provides the range of values of the thermal time constant of the system to be 0.6 ms to 5 ms.

At this point, all the model parameters were fit except the electrical and thermal capacitances. These parameters only affect the dynamics of the model; hence they could only be fit to the phase shift data. Since each evaluation of the compact model took >30 s, a manual fit to the last two parameters using a smaller subset of the data was attempted. The manual fit was considered sufficient, given the qualitative and quantitative accuracy of the reproduced phase shifts.

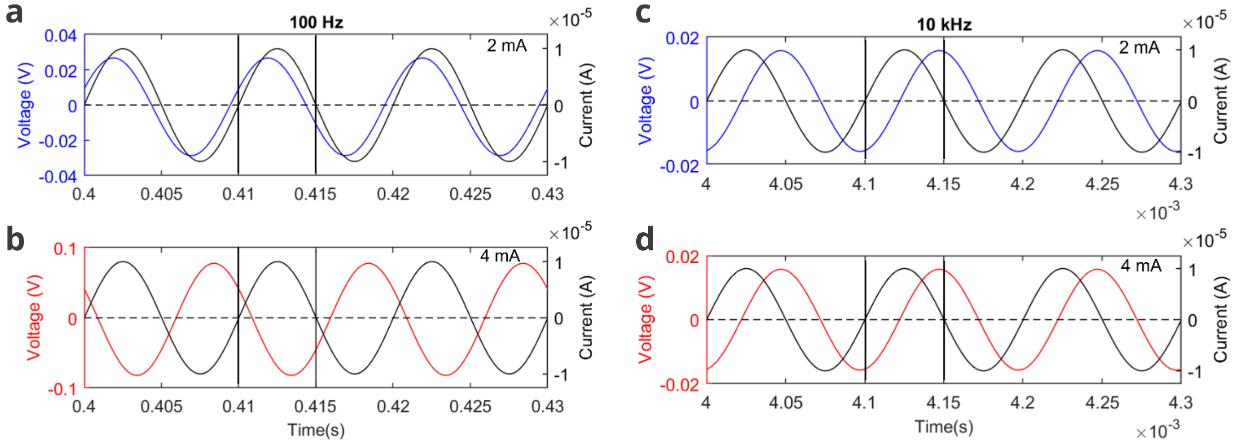

Supplementary Fig. 3: Simulated phase shifts at selected biases and frequencies. Phase shifts of voltage with respect to current from simulations of the model. (a) At a PDR bias of 2 mA at a frequency of 100 Hz. (b) At an NDR bias of 4 mA at a frequency of 100 Hz. (c) At a PDR bias of 2 mA at a frequency of 10 kHz. (b) At an NDR bias of 4 mA at a frequency of 10 kHz. The model reproduces bandwidth-limited super-quadrature phase shifts that occur only at the NDR bias, which we argue is semi-stable EoC.

2.3. Modeled super quadrature phase shifts

Once the compact model was fit, we evaluated it with a numerical solver to generate phase shifts for all experimental conditions in the main text (14 frequencies and 27 bias currents). Not only does the model

reproduce the super-quadrature phase shifts within the non-oscillating NDR region, but the generated phase shifts are also in good quantitative agreement with the experiments (Supplementary Fig. 3).

Simulated phase shifts were computed at all experimental biases and frequencies, showing good qualitative and overall quantitative agreement with the experimental phase shifts (Supplementary Fig. 4). The mean absolute error over all conditions was 0.06 (in units of 2π, i.e., 6% of the possible range). The maximum absolute error was 0.21 across the whole space of experimental conditions. Given the simplifying assumptions of the model, and that the thermal properties of the $LaCoO_3$ were never directly measured, this result is considered sufficient agreement to qualitatively verify the experimental observations of super-quadrature phase shifts.

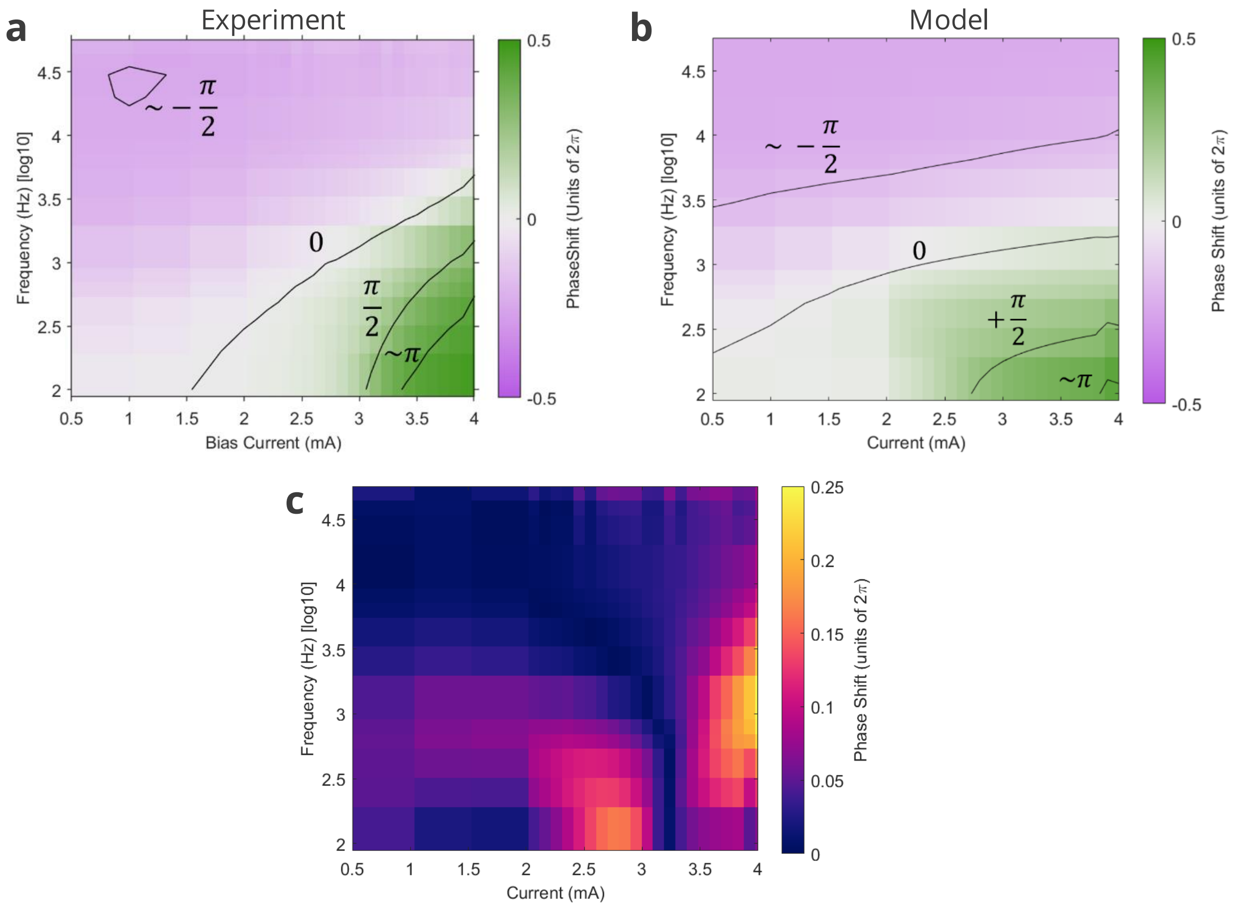

Supplementary Fig. 4: Summary of simulated phase shifts. (a) We have reproduced Fig. 3c in the main text , together with (b) a corresponding contour plot of simulated bias and frequency-dependent phase shifts. The simulated phase shifts exhibit general agreement with experiment, i.e., negative quadrature phase shifting at high frequency, and super-quadrature phase shifting at low frequencies and currents > 2.8 mA. (c) Quantitative comparison via absolute errors exhibits good agreement between the model and data.

3. Additional figures (referenced in the main text or in the Methods)

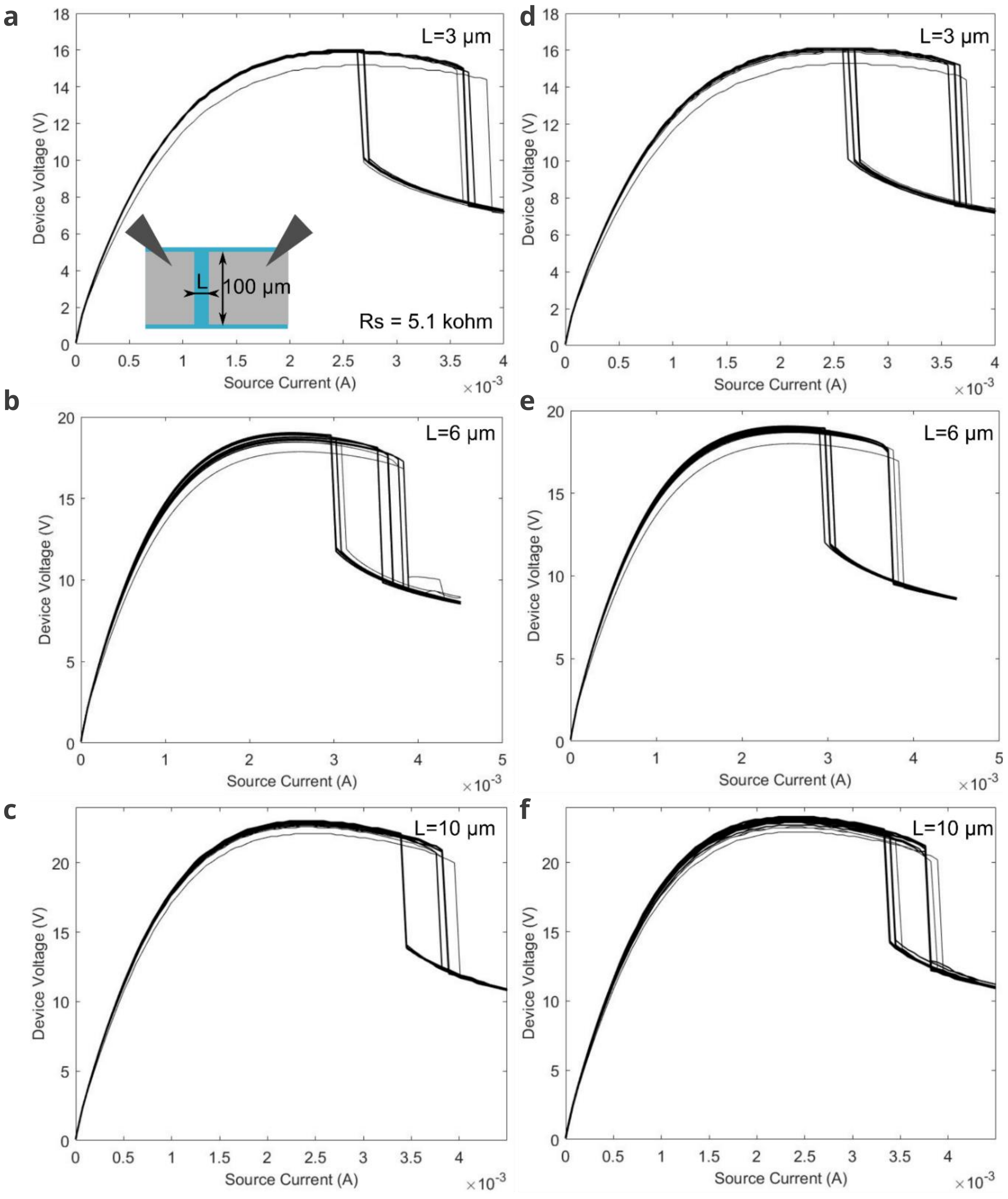

Supplementary Fig. 5: Cycle-to-cycle and device-to-device variation. (a)-(c) Repeated cycling of quasistatic IV sweeps on $LaCoO_3$ devices on the same chip with a constant 100 μm width and varied length. (d)-(f) Measurements similar to those in (a)-(c), repeated on identical replicates. All sweeps were taken using the same series 5.1 kΩ resistor for repeatability. Multiple cycles are overlaid, with the virgin (first) cycle shown as a grey line, with all subsequent cycles becoming gradually thicker.

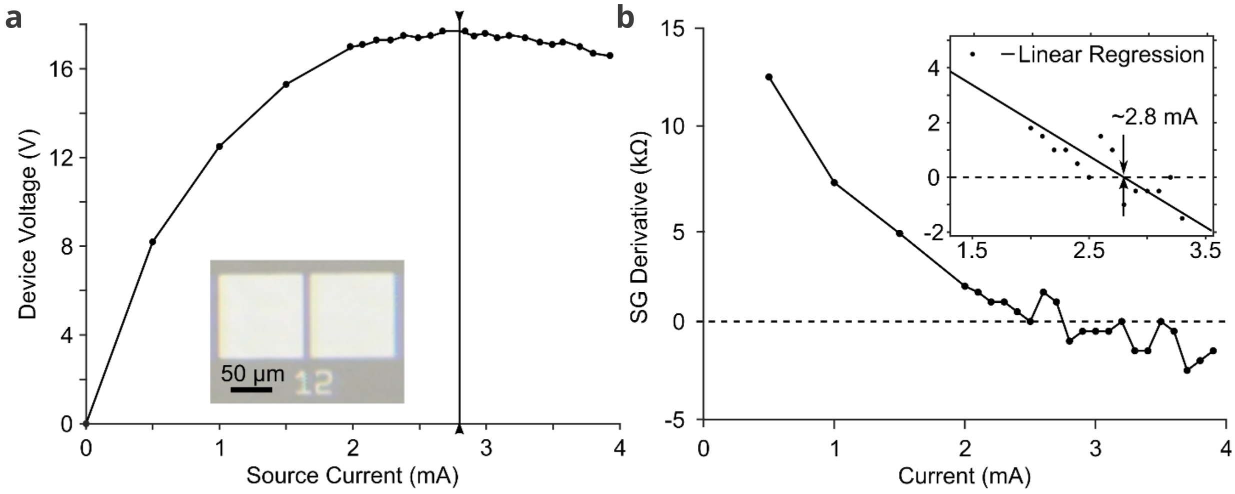

Supplementary Fig. 6: Calculation of NDR onset from device *IV* curve. (a) Reproduced *IV* curve from main Fig. 2c with an optical image of the two-terminal test structure (in the inset), with a vertical line marking the onset of NDR. (b) NDR onset computed by approximating derivative d*V*/d*I* with a Savitzky-Golay polynomial filter, then fitting a line to the filtered derivative data (in the inset) and finding its zero crossing.

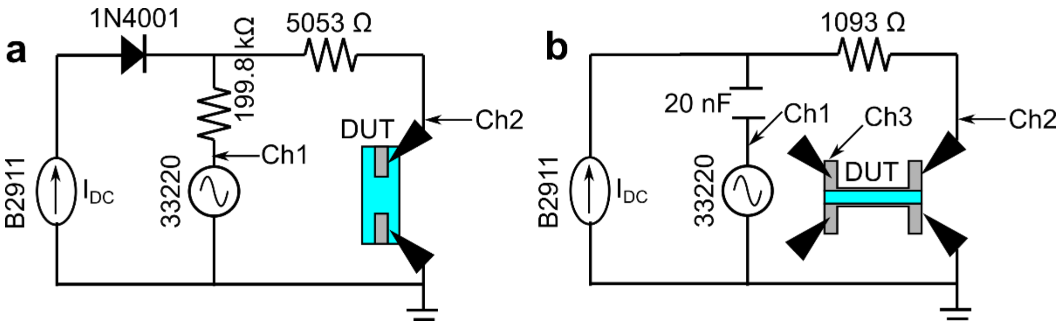

Supplementary Fig. 7: Test circuits for phase shifting and transmission line experiments. (a) For test structure, DC and AC sources were coupled through a diode to screen the SMU reactance from the function generator. (b) For the transmission line, similar coupling was obtained through a DC blocking capacitor.

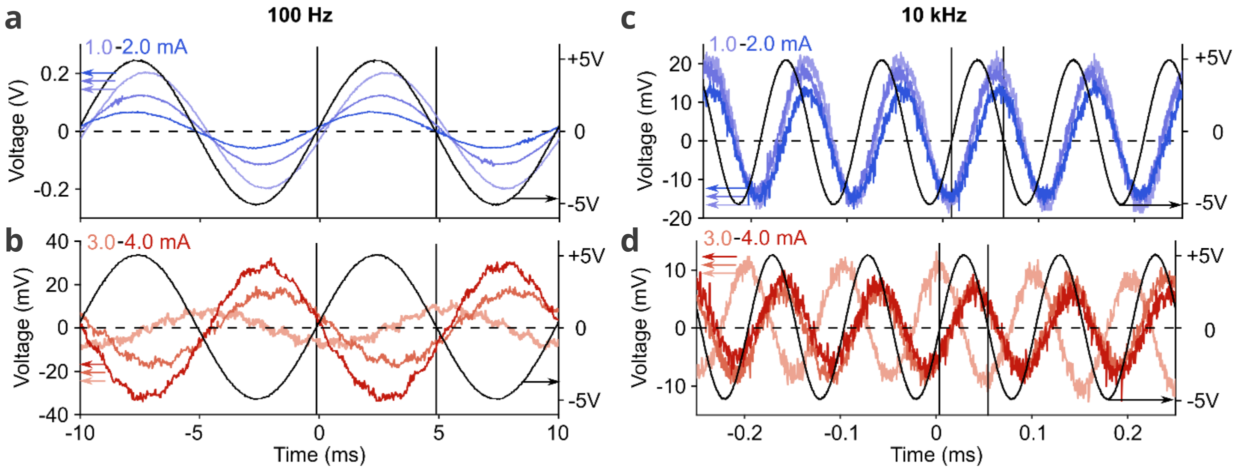

Supplementary Fig. 8: Raw sinusoid time series at selected biases and frequencies. Phase shifts of $LaCoO_3$ device voltage output (blue / red lines) relative to the fixed function generator input (black lines) as a function of biases. (a) In PDR bias at 100 Hz. (b) In NDR bias at 100 Hz. (c) In PDR bias at 10 kHz. (d) In NDR bias at 10 kHz. Zero crossings of sinusoids centered on the input maxima are marked with vertical black lines. At 100 Hz (a-b) the time series are strongly bias-dependent. For bias currents well below the NDR onset at about 3 mA (a, c), all of the phase shifts are sub-quadrature $|\Delta\phi| < \pi/2$. We emphasize this graphically by marking a $-\pi/2 \leq \Delta\phi \leq \pi/2$ region for the input data (vertical black lines); then for biases 1.0-2.0 mA, the nearest output maxima all fall within this region. Once the bias increases past the NDR onset at about 3 mA (b), the nearest output maxima move outside of the vertical lines (or equivalently, the nearest minima lie within the lines), meaning the phase shifts have become super-quadrature. At 10 kHz (100× higher frequency, c-d), these results are different. For the entire bias range before and after the NDR onset, the phase shifts are always sub-quadrature. In (d), $\Delta\phi \approx -\pi/2$ for a bias of 3 mA, consistent with main Fig. 3c. These observations verify that the super quadrature phase shifting is bandwidth-limited, in agreement with SI section 1.

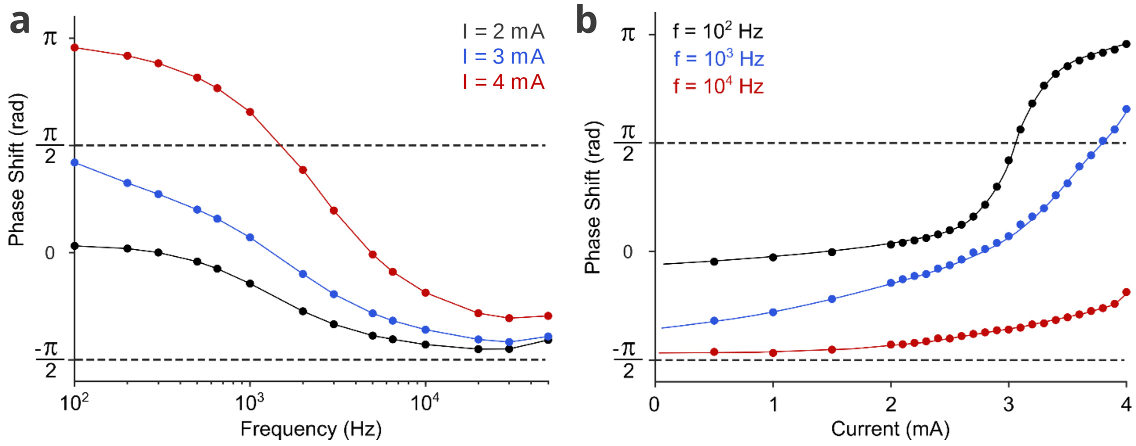

Supplementary Fig. 9: Selected cross-sections of the contour phase shift plot (main Fig. 3c). (a) Measured phase shifts as a function of frequency at selected bias currents and, (b) of bias current at selected frequencies.

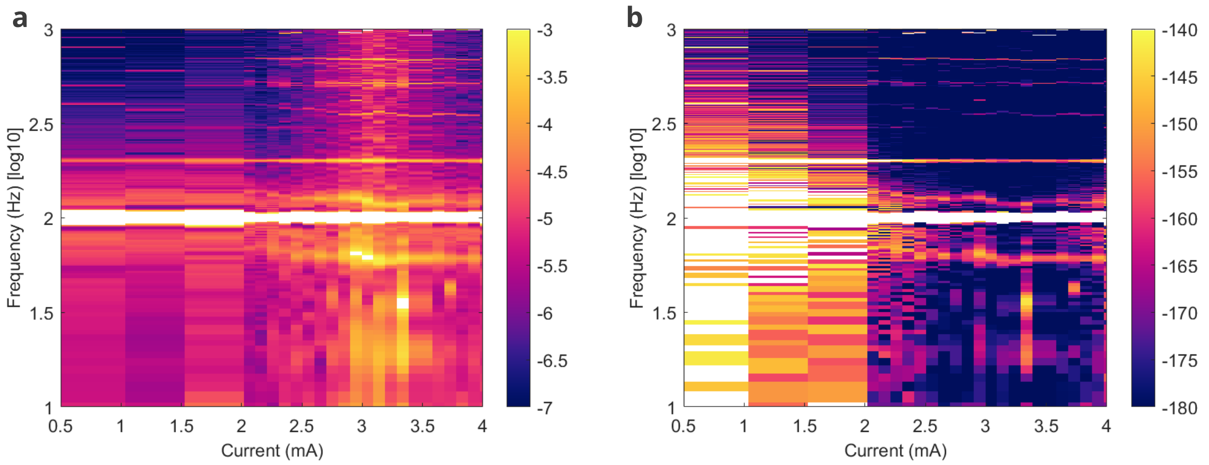

Supplementary Fig. 10: Effect of Johnson Nyquist normalization on amplification data. Normalized data (from main Fig. 3d). (b) Raw data with Johnson Nyquist scaling obscuring the amplification peak.

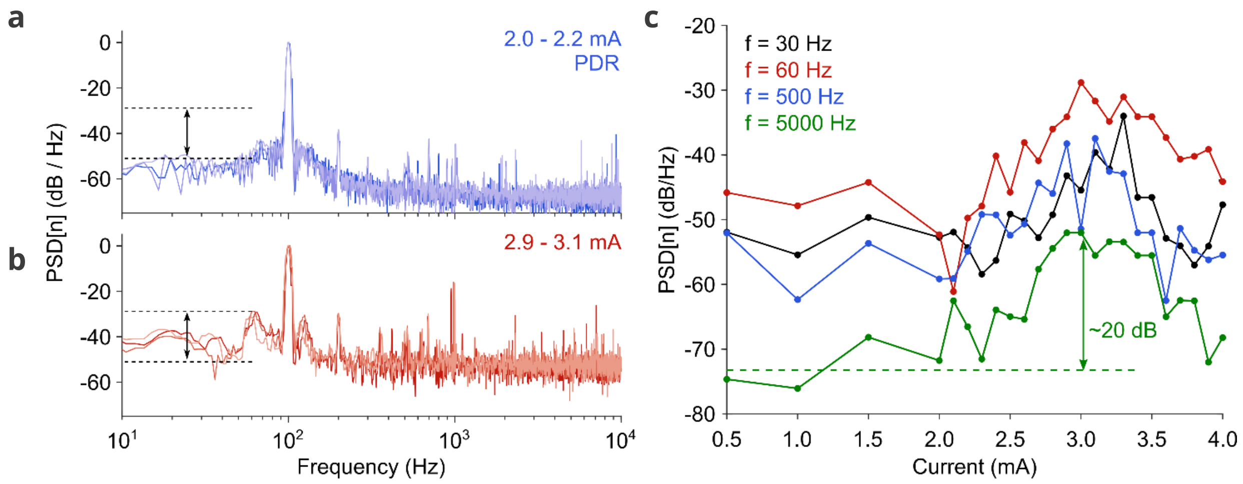

Supplementary Fig. 11: Selected cross-sections of the power spectral density contour plot. (a)-(b) Power spectral densities normalized by 100 Hz component, as a function of frequency at fixed bias currents, and (b) at fixed frequencies. The relative noise at essentially all frequencies increases by ~100x as the bias current approaches the NDR onset.

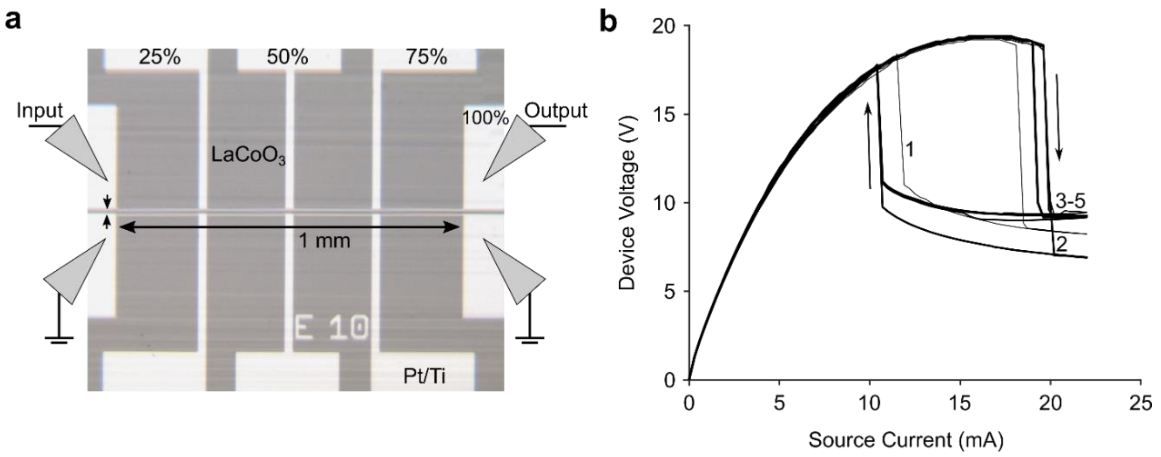

Supplementary Fig. 12: Device structure and *IV* sweep for the 1 mm transmission line. (a) Optical image of the 1 mm transmission line structure with intermediate measurement pads (25%, 50%, 75%). (b) Five consecutive *IV* sweeps measured across the left-most pads.

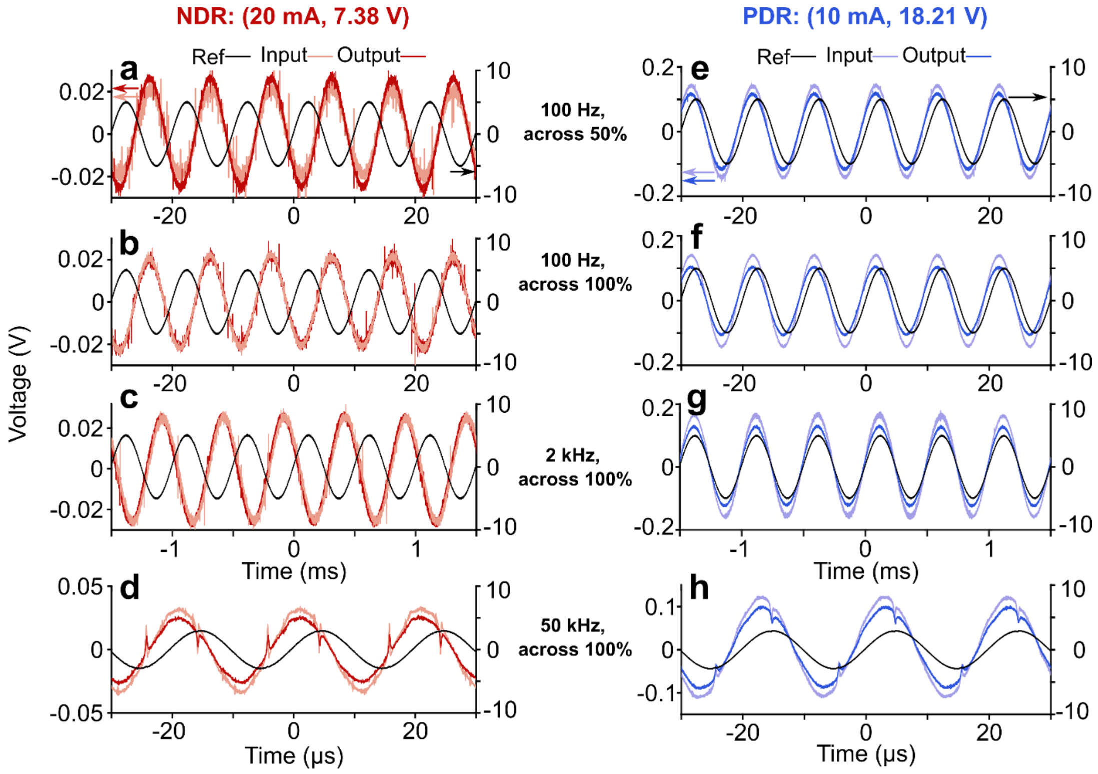

Supplementary Fig. 13: Raw sinusoids for the 1 mm transmission line under various conditions. (a) Reference signal from function generator (black), transmission input (light red), and 50% transmission output (dark red) at 100 Hz, when DC biased in the NDR region; (b) same for transmission across 100% length at 100 Hz, (c) 100% transmission at 2 kHz, (d) 100% transmission at 50 kHz, (e-h) corresponding data for transmission line DC biased in the PDR region. No active transmission with greater than unity gain is observed in the PDR region.

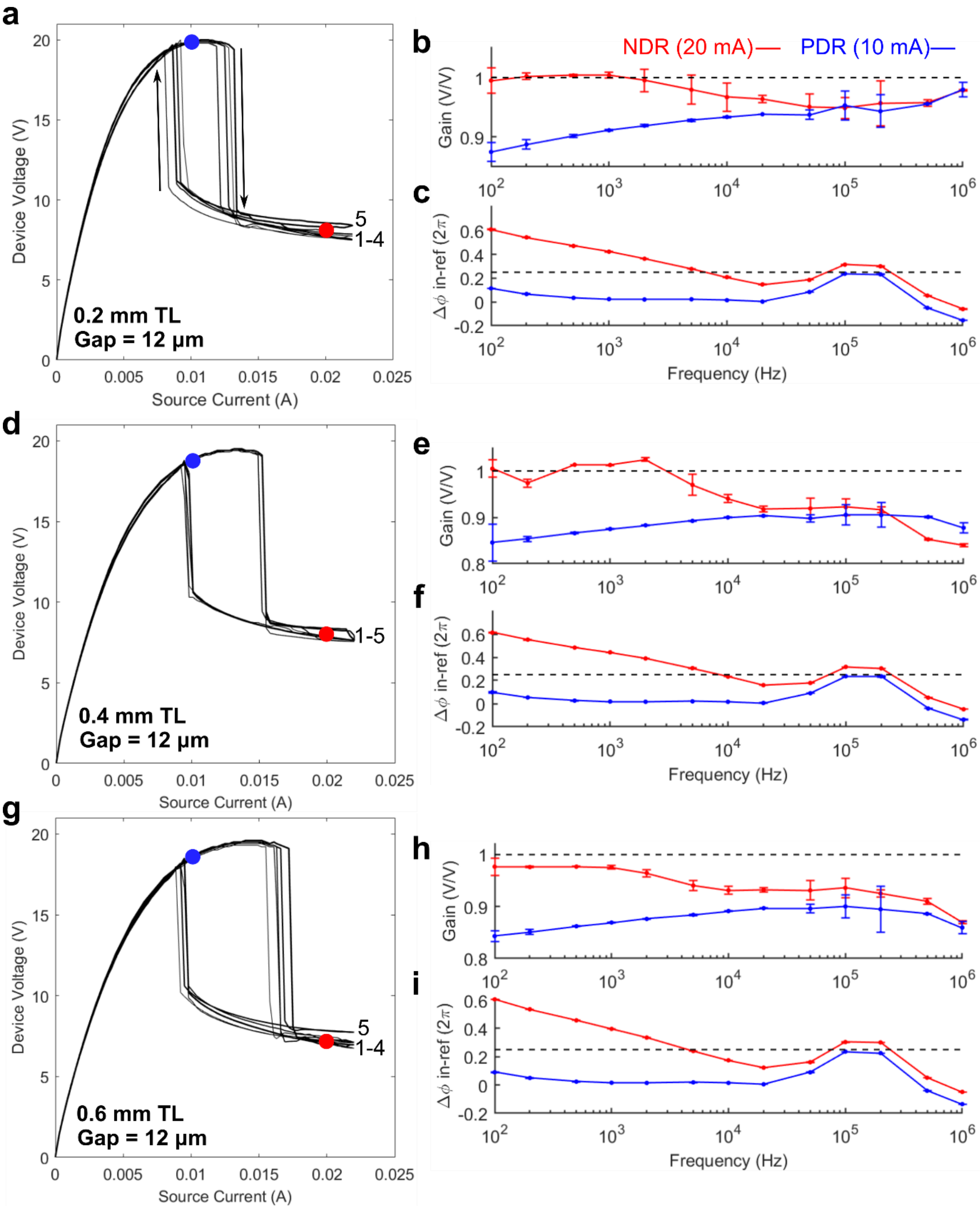

Supplementary Fig. 14: Dependence of active transmission on transmission line length. For transmission line with 0.2 mm length, (a) five consecutive quasi-DC current-voltage sweeps, (b) frequency dependent gain of output vs. input across total 0.2 mm length, (c) frequency dependent phase shift between function generator and transmission line input displaying super-quadrature phase shifting in

NDR, (d-f) corresponding data for 0.4 mm transmission line, (g-i) corresponding data for 0.6 mm transmission line.

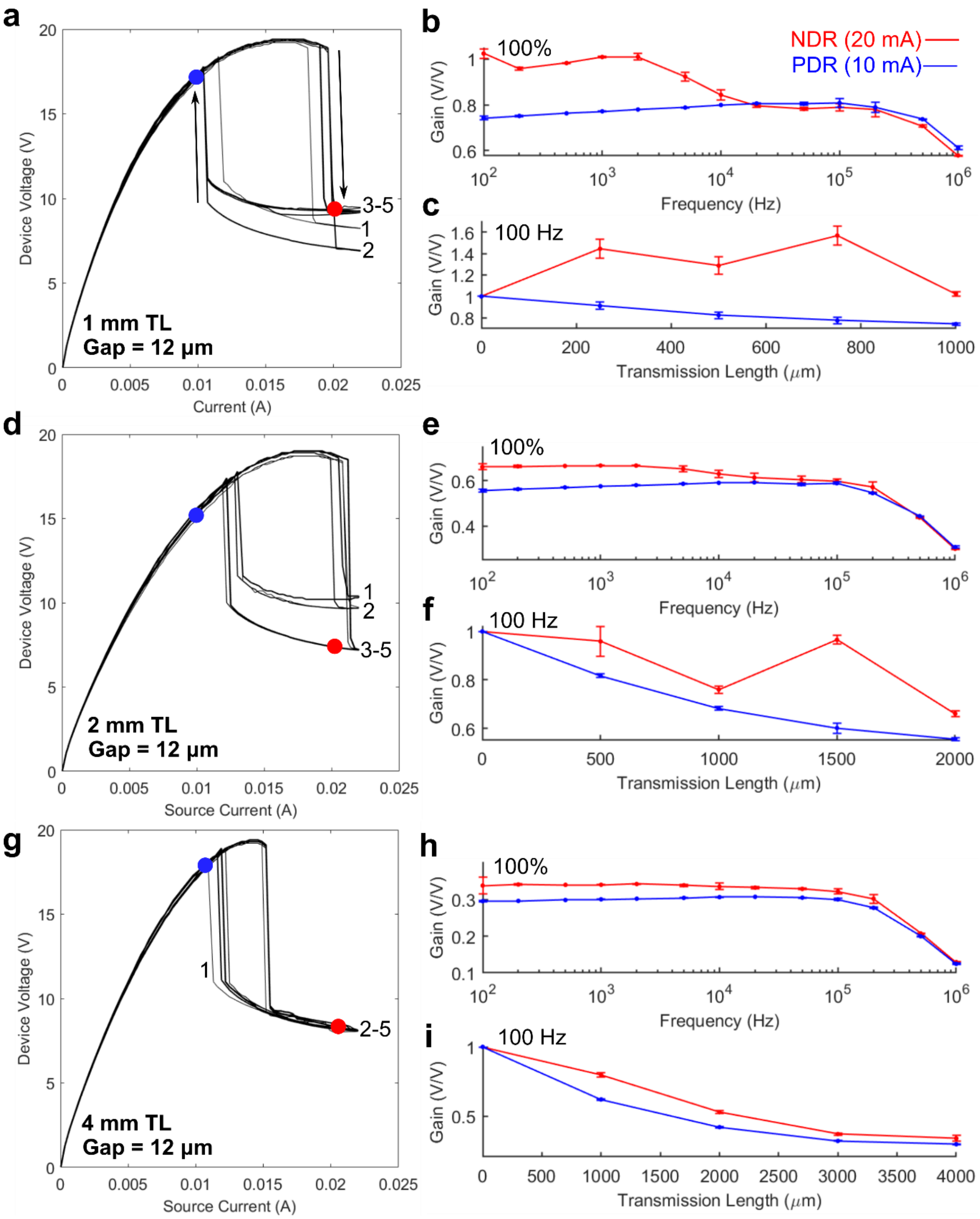

Supplementary Fig. 15: More transmission line length dependence and intermediate transmission. For transmission line with 1 mm length, (a) five consecutive quasi-DC current-voltage sweeps, (b) frequency dependent gain of output vs input across total 1 mm length, (c) length dependent gain at

intermediate transmission points 25%, 50%, and 100% across total length, (d-f) corresponding data for 2 mm transmission line, (g-i) corresponding data for 4 mm transmission line.

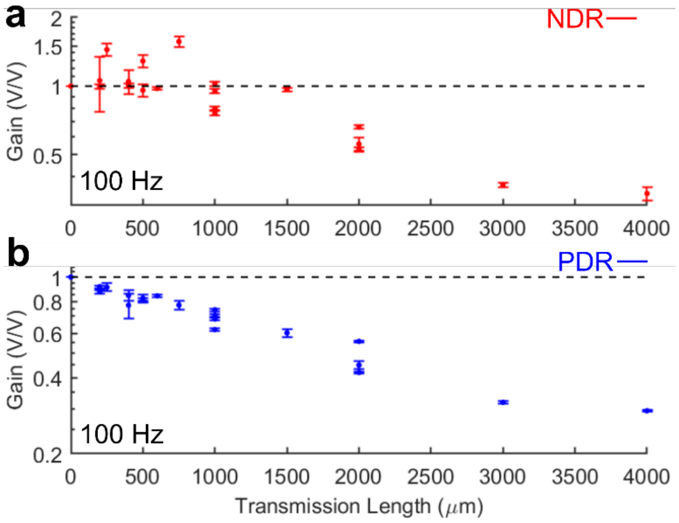

Supplementary Fig. 16: Compiled transmission line gains at 100 Hz. (a) Compiled transmission line gain data for transmission lines with 10 μm and 12 μm gap size, and 0.2 mm – 4 mm length, when biased variously in the NDR region; (b) corresponding compiled data for transmission lines biased in the PDR region. The vertical axis is in logarithmic scale. Clearly, the gain drops exponentially with distance in the PDR region, as expected.

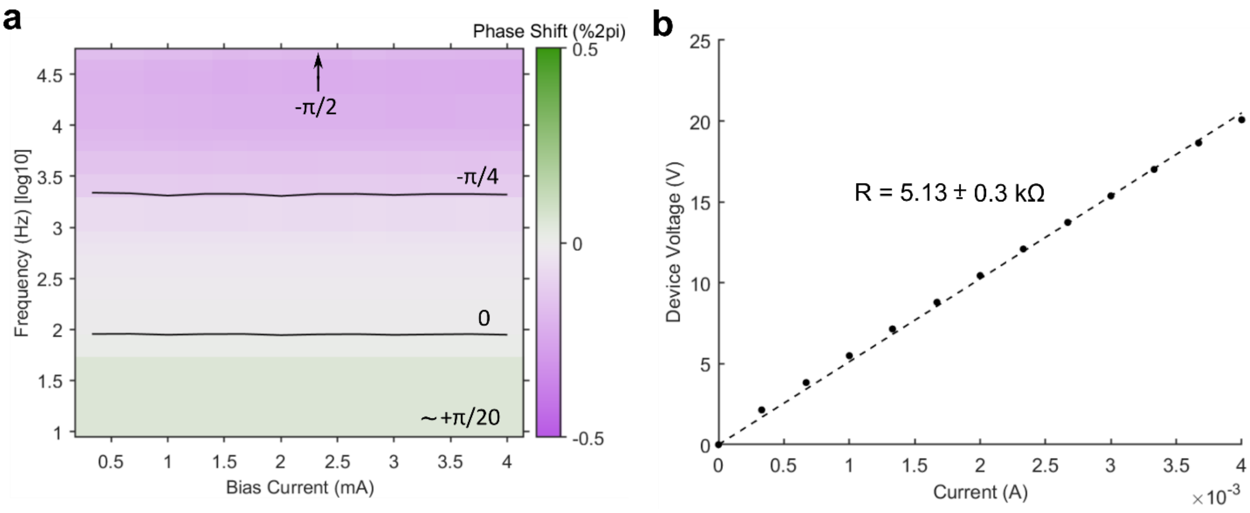

Supplementary Fig. 17: Absence of super-quadrature phase shifts for a passive 5.1 kΩ resistor. (a) Current bias-dependent and frequency-dependent current-voltage phase shifts for test circuit with an off-the-shelf resistor. (b) Corresponding quasi-DC *I*-*V* sweep.

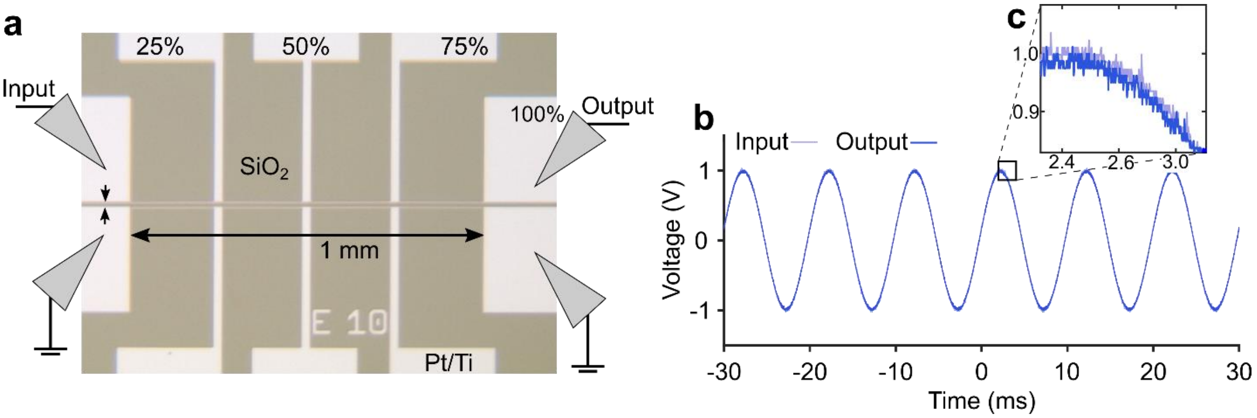

Supplementary Fig. 18: Control data for passive SiO2 transmission line. (a) Optical image of 1 mm transmission line structure on SiO_2 (passive medium). (b) Raw input and output sinusoid data (c) Zoomed in inset of data shows slight attenuation. No active transmission or amplification was present under any operating condition.

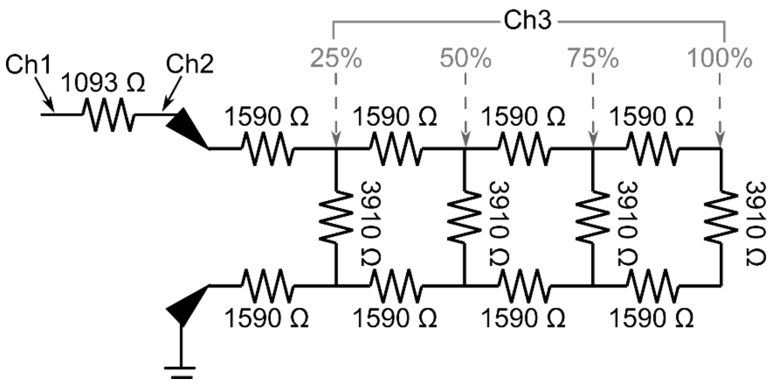

Supplementary Fig. 19: Discretized model of a passive transmission line. To validate the test circuit and provide a comparison with active transmission data, a four-cell discretized “transmission line” was constructed from off-the-shelf discrete passive resistors. Resistor values were determined to be representative of 1 mm transmission line in the main text.

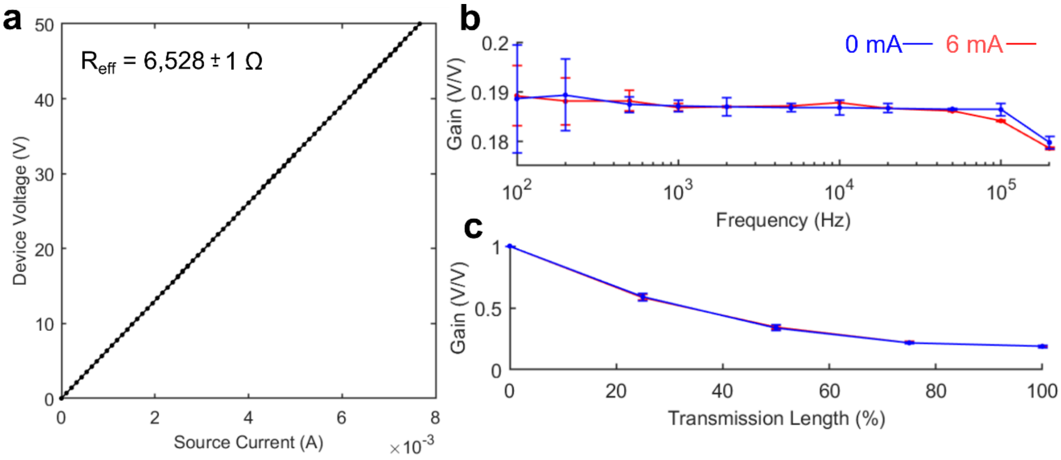

Supplementary Fig. 20: Control data for discrete resistor model of passive transmission line (Supplementary Fig. 19)**.** For transmission network composed of passive resistors, (a) two consecutive quasi-DC current-voltage sweeps, (b) frequency dependent gain of output vs. input across the total network, (c) length dependent gain at intermediate transmission points 25%, 50%, and 100% across network.

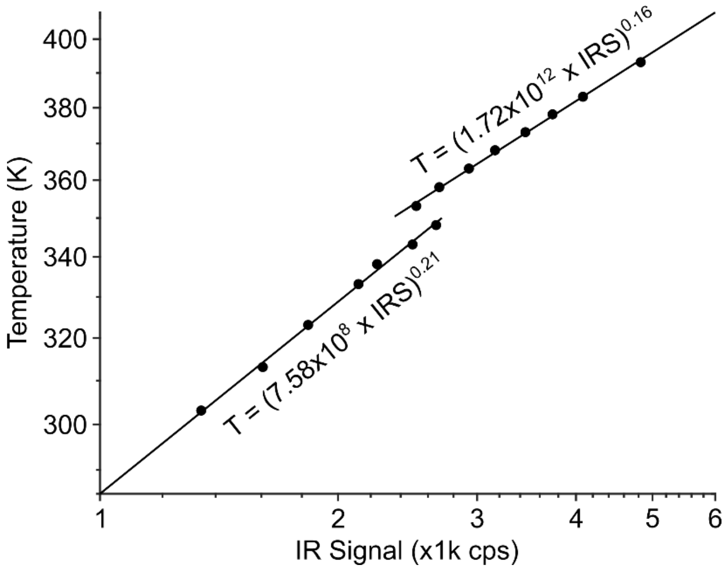

Supplementary Fig. 21: IR Camera Calibration. IR camera data were calibrated by using the camera to record large areas of $LaCoO_3$ blanket film while heating with a temperature stage, and plotting the recorded IR intensity versus the temperature. A piecewise calibration curve was fit according to a power law $T = \alpha(IR)^{\beta}$. cps: counts per second.

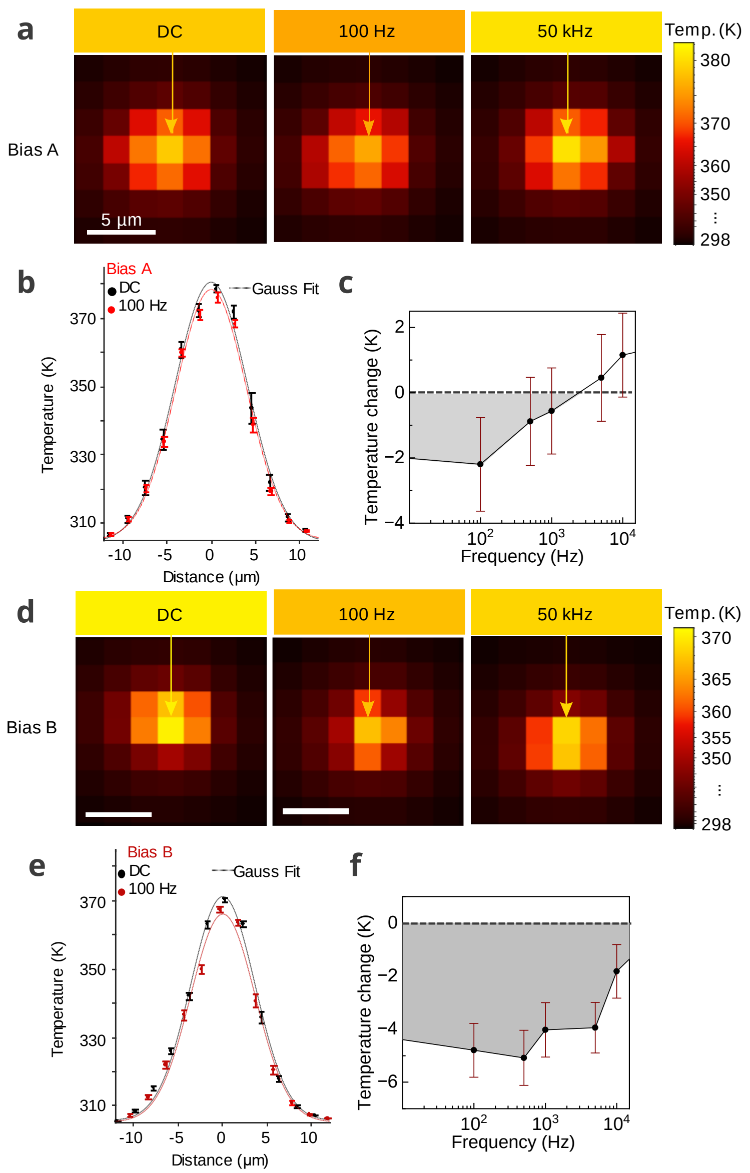

Supplementary Fig. 22: Additional thermal maps at another bias. (a)-(c) are replotted from main Fig. 5a-c. These data were obtained at Bias A (3 mA), which was a bias just after the onset of the NDR. (d)-(f) are data similar to (a)-(c), but at Bias B (4.1 mA), which was a bias well after the onset of NDR. In Bias B, the frequency range for which there is localized cooling (i.e., energy for amplification) is much higher (beyond 10 kHz), whereas for Bias A, this limit is just about 1 kHz. Thus, the bandwidth for amplification can be tuned to a significant extent by altering the applied bias.
